# Mammography screening status of women aged 40 and older in eastern Iran using the precaution adoption process model (PAPM)

**DOI:** 10.1038/s41598-025-08511-3

**Published:** 2025-07-13

**Authors:** Mahbobe Sadat Sahebi, Gholamreza Sharifzadeh, Mitra Moodi

**Affiliations:** 1 Health Education and Health Promotion, Student Research Committee, School of Health, Social Determinants of Health Research Center, Birjand University of Medical Sciences, Birjand, Iran; 2 Department of Epidemiology and Biostatistics, School of Health, Social Determinants of Health Research Center, Birjand University of Medical Sciences, Birjand, Iran; 3 Department of Health Education and Promotion, School of Health, Geriatric Health Research Center, Birjand University of Medical Sciences, Birjand, Iran

**Keywords:** Precaution adoption process model (PAPM), Breast cancer, Mammography, Screening, Prevention, Women health, Medical research, Breast cancer, Cancer screening

## Abstract

Considering the importance of mammography in reducing breast cancer mortality, this study was conducted with the aim of determining the status of mammography and related factors based on the Precaution Adoption Process Model (PAPM) in women 40 years of age and older. In Iran, breast cancer is the most common cancer among women, with an age-standardized incidence rate of 43.02 per 100,000 in 2017. The descriptive-analytical study was conducted on 354 women aged 40 and older referred to comprehensive health service centers in Gonabad city (Eastern Iran) in 2023. To determine the stages of decision-making, a researcher-made questionnaire based on the PAPM was used, and people were classified in the stages of unaware, unengaged, deciding to act, decided not to act, decided to act, acting and maintenance. The data was collected by telephone interview and analyzed by statistical tests. The mean age of women was 53.55 ± 7.8 years. In terms of decision-making stages, 19.8%, 39.5%, 9.9%, 21.5%, 9.3% in the unaware stage, unengaged, decided not to act, decided to act, acting and maintenance of mammography screening behavior. Statistical tests showed that the variables of disease history (*p* < 0.0001), family history of disease (*p* < 0.0001), education level (*p* = 0.021) and BMI (*p* = 0.003) with the stages of intention to perform mammography had a significant relationship. The findings showed that most of the women were in the stage of unaware and deciding to act, and a small percentage were in the stage of decided to act acting and maintenance. Therefore, it seems necessary to carry out educational interventions based on decision-making processes in order to improve mammography screening behavior and early diagnosis of breast cancer.

**Supplementary Information:**

The online version contains supplementary material available at 10.1038/s41598-025-08511-3.

## Introduction

**Fig. 1:**
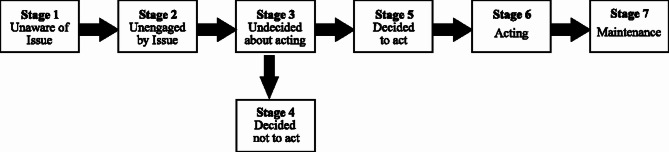
Stages of the precaution adoption process model.

Breast cancer is one of the most common types of cancer worldwide^1,2^. Estimates show that approximately one in five men or women will develop cancer in their lifetime, while one in nine men and one in 12 women will die from it. Lung cancer was the most common cancer diagnosed in 2022, responsible for approximately 2.5 million new cases or one in eight cases of cancer worldwide (12.4% of all cancers globally), followed by Female breast (11.6%) and colon cancer (9.6%) are located^3^. Every year, more than one million and one hundred thousand women in the world are diagnosed with breast cancer^4^. In Iran, the results of an epidemiological survey of breast cancer showed that most cases of breast cancer (82%) are diagnosed in the final stages. The incidence of breast cancer showed an increasing trend in Iran according to national cancer registry reports between 2003 and 2017 in both women and men. In 2003 and 2017, the age-specific incidence rates of breast cancer were 15.96 and 40.72 per 100,000 women, respectively. In 2017, the highest incidence rate of breast cancer was observed in the age groups of 65–69 years (128.33 per 100,000 women) and 60–64 years (127.79 per 100,000 women) and mostly in Isfahan, Yazd, Gilan, and Alborz provinces^5^ Also, Iranian women with breast cancer are 10 years younger than their counterparts in western countries, and often present with advanced stages of cancer^6,7^. Early detection of breast cancer can lead to almost complete treatment, and with timely diagnosis and effective treatment, the survival rate reaches 90%^8^. In high-income countries such as the United States and Canada, the incidence rates of some cancers, such as breast cancer and thyroid cancer, have increased in recent years, in part due to better access to or screening, according to recent data^9^. Mammography is a special type of imaging that is commonly and increasingly used for preparing accurate images of the breast and screening and early detection of breast cancer in the early stages^10^. Screening mammography aims to identify breast cancer at earlier stages of the disease, when treatment can be more successful^11^Research shows that the use of mammography reduces the death rate caused by breast cancer, so the death of 1% of women aged 50 to 70 can be postponed by performing this screening test^12^. In developing countries, women between the ages of 50 and 74 are usually recommended to have regular mammograms every 2 or 3 years^1^ Today, breast cancer deaths have decreased, and among the factors of this reduction, mammography screening has been able to reduce the death rate of breast cancer by 25 to 35% due to its ability to detect the disease in its early stages. However, barely one third of women over 50 years of age perform screening mammography^13^. Considering the high prevalence of breast cancer in Iranian women and the specificity of mammography in breast cancer diagnosis, it seems necessary to encourage women to perform mammography. Encouraging women to perform mammography requires a change in their attitude and behavior, and to change people’s behavior, it is necessary to know their opinions and motivation^14^. Behavioral science can be used to better understand different types of decision making for behaviors such as participating or not participating in cancer screening programs. For example, a person may never have been screened or may have been screened but not as recommended. In both groups, the motivations may also be different. People may not know that they should be screened, so they avoid screening or consider preparing for screening^15^. The Precaution Adoption Process Model (PAPM) explains how people adopt certain behaviors to take care of themselves. The concepts of this model were first proposed in 1988 by Weinstein, then was further refined in 1992 by Weinstein and Sandman that suggested seven behavioral stages in PAPM ranging from unaware of issue, unengaged by issue, deciding, decided not to act, decided to act, acting and maintenance^16,17^. (Fig. 1)

In Iran, breast cancer screening programs face significant challenges, including low participation rates. A study conducted in Arak reported that only 38.5% of women aged 30 to 70 participated in breast cancer screening programs^18^. In comparison, in high-income countries like the United States, the participation rate in breast cancer screening programs varies by region, but it has been reported that approximately 70% of eligible women participate in mammography screenings^19^. However, this rate has seen a slight decline in recent years due to various barriers such as lack of access to healthcare services, awareness gaps, and socioeconomic factors. These differences emphasize the urgent need for improved public awareness, better access to screening services, and more effective informational campaigns in Iran to encourage greater participation in breast cancer screening^18^. Stage models assume that people go through a progression of thoughts or stages when considering a health behavior and ultimately taking action^20^. PAPM suggests people move through a series of steps toward participating in cancer screening. It highlights the role of past behavior and differentiates between motivations for non-attendance, including conscious decisions not to participate. It also acknowledges the importance of turning intention into action. Several factors influence the progression through these stages, including previous health behaviors, motivation, awareness, and perceived barriers^21^. This model has been used in colorectal cancer screening^15,22,23^Also in cancer screening, PAPM to identify decision-making stages of people related to breast cancer screening^21^ and cervical cancer screening^23–26^ has been used. According to this model, not all non-users are the same, and determining where they are in the decision-making process should allow researchers to design more effective interventions. Recognizing that not all individuals start at the same decision-making stage and therefore may not have the same intervention needs can help improve the effectiveness of intervention efforts^20^. And since women are an important part of society whose health is deeply linked to society’s health, it is essential to measure their awareness and motivation of preventive behaviors with the aim of effective educational planning^27^.

Considering the growing trend of breast cancer in Iran and the fact that many patients with breast cancer refer to the advanced stages of cancer, the need to reflect and address this problem in order to improve the behavior of breast cancer control and intervention by implementing the model It seems important to change behavior.

The population under investigation is women over 40 years of age who refer to the health care centers of Gonabad city, and considering that so far, no research has been done regarding the behavior of mammography in the community of women referring to the health care centers of Gonabad city based on the Precaution Adoption Process Model. The present study was conducted with the aim of determining the status of mammography and its related factors based on the Precaution Adoption Process Model in women over 40 years of age who referred to comprehensive health services in Gonabad (Eastern Iran).

## Methods

### Study design and participants

In this descriptive-analytical cross-sectional study, 354 women over 40 years of age visiting health centers in Gonabad to receive various health services in 2023–2024 participated. The sample size was based on the objective of determining the frequency distribution of the stages of behavior change in the studied women and based on the results of the study by Moody et al.^28^ and with a reliability coefficient of 0.95 and alpha equal to 0.05, and using this formula $\:\text{n}=\frac{{({z}_{1-\frac{a}{2}})}^{2}*P\left(1-P\right)}{{\text{d}}^{2}}354$, the sample size was estimated to be 354 people.

### Sampling

The sampling method was a multi-stage cluster. In this way, first comprehensive urban health centers were considered as clusters. In each cluster, the list of women eligible to enter the study in each center was extracted based on the apple system and systemically randomly extracted from the list, and the sample size in each center was selected according to the total number of calculated samples. The researcher by telephone for inclusion and exclusion criteria and briefly informed the eligible ones of the research objectives.

The inclusion criteria were as follows:

- having at least 40 years of age under the coverage of comprehensive health service centers in Gonabad (Eastern Iran), and not having any history of breast cancer.
- Also, no cancer diagnosis was reported for any of the research samples.
- Lack of employment in professions related to health and treatment, have minimum literacy and informed consent to participate in the study.

Study exclusion criteria:

- Women under 40 years of age.
- Women who were unwilling to participate in the study.

### Data collection tools

Data collection was done by means of a researcher-made questionnaire, including three parts: demographic information, awareness questions, and questions related to the stages of the mammography behavior based on the PAPM; which included 15, 22 and 7 questions respectively. The way of scoring the knowledge questions was that the correct answer was scored as 2, the I don’t know option as 1, and the wrong answer as 0.

Based on the literature review, the initial questionnaire was designed to determine the validity of the content by 15 professors and their opinions were applied in the questionnaire, and the values of CVI and CVR were obtained as 0.87 and 0.7 respectively.

Also, the reliability of the data collection tool based on the Test-Retest method was conducted on 20 people similar to the study participants at an interval of two weeks, and the correlation was 95%.

### Data analysis

After collecting the data, the data were analyzed by SPSS version19, and mean, standard deviation, and frequency distribution tables were used to describe the data. The normality of the quantitative data was first confirmed by evaluating the skewness and kurtosis and visual charts and the Kolmogorov-Smirnov test. Then, descriptive statistics of frequency (percentage) and mean (SD) were used to investigate the behavior of mammography screening and the level of awareness of the participants, and independent *t* test, and backward regression analysis were used to analyze the data. The significance level was set at 0.05.

### Ethics approval and consent to participate

The current study received approval from the Research Vice-Chancellor and the Ethics Committee at Birjand University of Medical Sciences under the code IR.BUMS.REC.1402.323. Initially, the objectives of the research, participant anonymity, voluntary involvement, and study details were verbally communicated. Subsequently, these were read and acknowledged through a signed written informed consent form. All methods were performed in accordance with the relevant regulations and guidelines according to the Declaration of Helsinki.

## Results

In this study, 354 women 40 years of age and older in Gonabad city (Eastern Iran) were investigated. The age range of the studied subjects was 40 to 70 years with an average and standard deviation of 53.55 ± 7.8 years.

The majority of the studied subjects were married, 97.5%, 56.2% postmenopausal, and 38.4% had secondary education. In terms of the stages of decision to perform mammography screening behavior, the results showed that most of the studied subjects were in the stage of non-involvement and the least were in the stage of screening. (Table 1)

**Table 1:** Demographic characteristics of the study subjects.

Variable		Number	Percentage
Marital status	Married	345	97.5
	Single	9	2.5
	Total	354	100
Menopausal status	Yes	199	56.2
	No	155	43.8
	Total	354	100
Breastfeeding status	Yes	344	97.2
	No	10	2.8
	Total	354	100
Education level	Primary	31	8.8
	Secondary	112	31.6
	Diploma	136	38.4
	University	75	21.2
	Total	354	100

Chi-square test showed a significant difference in the decision-making stages of mammography according to the history of the disease and the family history of the disease. (Table 2) The results of the chi-square test also showed that there was a significant difference in the stages of mammography intention according to education (Table 3), but this test did not show a significant difference between the stages of intention to perform mammography behavior with job and insurance status.

**Table 2:** Frequency of people according to decision-making stages (354 Participants).

Stages	Number	Percent
Stage1 (unaware of issue)	0	0
Stage2 (unengaged by issue)	70	19.8
Stage3 (undecided about acting)	140	39.5
Stage4 (decided not act)	35	9.9
Stage5 (decided to act)	76	21.5
Stage6 (acting)	33	9.3
Stage7 (maintenance)	0	0
Total	354	100

**Table 3:** Decision stages according to the disease and family history of the disease in the study.

		Stage					Total	P
		Stage2 (unengaged by Issue)	Stage3 (undecided about acting)	Stage4 (decided not act)	Stage5 (decided to act)	Stage6 (acting)		
Sickness	Yes	1	6	0	14	7	28	< 0/0001^*^
	No	69	134	35	62	26	326	
History of family disease	Yes	6	27	4	37	20	94	< 0/0001^*^
	No	64	113	31	39	13	260	

*P-value < 0.05 is significant.

The results showed that as the age of the participants in this study increased, the level of awareness of mammography screening behavior was also higher (*p* < 0.005).

The women participating in the study who were in the stage of decision to perform the screening behavior had a higher awareness score than those who were in the non-conflict and non-decision stages of performing mammography. The results of one-way analysis of variance showed that there is a significant relationship between participants’ knowledge and education. (B = 1.4 and *P* < 0.0001) The regression results showed that there is a significant relationship between awareness and education. (B = 1.4 and *P* < 0.0001). The results of the study showed that people with lower education are in the initial stages of deciding to perform a mammography screening test (Table 4). In addition, regression analysis showed that there is a relationship between age (B = 0.18 and *P* = 0.04) and disease history (B = 0.7 and *P* = 0.004) and family history (B = 0.91 and *P* < 0.0001) there is a significant relationship (Table 5).

**Table 4:** Decision-making stages of mammography behavior according to education in the Participants.

Stage education	Stage2 *N* (%)	Stage3 *N* (%)	Stage4 *N* (%)	Stage5 *N* (%)	Stage6 *N* (%)	*P*
Elementary	9 (12.85)	9 (6.43)	7 (20)	2 (2.63)	4 (12.12)	0.023^*^
Secondary	24 (34.28)	49 (35)	11 (31.43)	21 (27.63)	7 (21.21)	
Diploma	24 (34.28)	59 (42.14)	13 (37.14)	28 (36.84)	12 (36.36)	
University	13 (18.57)	23 (11.43)	4 (11.43)	25 (32.90)	10 (30.30)	
Total	70 (100)	140 (100)	35 (100)	76 (100)	33 (100)	

^*^P-value < 0.05 is significant.

**Table 5:** Backward regression adjustment for factors related to stages of mammography screening in women.

Variable	B	Std. error	Beta	*p*-value
Age∗	0.019	0.008	0.115	0.021^**^
Sickness	0.691	0.243	0.147	0.005^**^
Family disease	0.906	0.151	0.314	0.000^**^

*Women over 40 years old; ^**^P-value < 0.05 is significant.

## Discussion

The present study was conducted with the aim of investigating the status of mammography and its related factors based on the precautionary acceptance process model in women 40 years of age and older referring to comprehensive health service centers in Gonabad city (Eastern Iran).

In this study, women who had never been screened for breast cancer made up more than half of the participants, who were at different stages. These results indicate the importance of a differentiated approach in considering the adoption stages. These results were consistent with the study of Sorai Choi who conducted a study on Korean women^29^. But these results were not consistent with Kang Min-Jung’s study titled Integrating the Precautionary Acceptance Process Model and the Health Belief Model, which she conducted to assess cancer screening readiness among Korean adults. In this study, 29.8% of the people participating in the study were in the operation stage^30^. And this is despite the fact that in the present study only 9.3% of people were in the operation stage.

In this study, people who had the intention to perform mammography screening (step five) formed the second group in terms of having the highest percentage of people. Despite the intention to perform the behavior, they had not yet reached the stage of action (Stage 6). This gap between intention and actual behavior is a well-established concept in behavioral sciences. Many studies have demonstrated that while intention to act is common, many individuals fail to follow through with the intended action. Previous studies have shown that intending to perform a behavior but not achieving it is one of the most commonly endorsed reasons for not engaging in preventive health behaviors, and this gap between intention and behavior has long been recognized in the behavioral sciences and has existed before. Similar results were observed in the study of Laura Marlow et al.^15^ Modi et al.‘s study^28^ on women teachers in Isfahan, Heydari et al.‘s study^31^ on women in southwestern Iran, showed that there was a significant relationship between the awareness of breast cancer screening and the level of education, which was consistent with the present study.

However, to address this gap between intention and action, it is essential to implement targeted health campaigns that focus not only on increasing awareness but also on encouraging actual behavior. One approach could involve organizing community-based educational programs and mobile health units to improve access to screening services, particularly in rural and underserved areas. Additionally, collaboration with local healthcare providers to offer incentives and reminders could further motivate women to follow through with their intentions.

One possible explanation for this gap is that despite a clear intention to perform a behavior like mammography, factors such as fear, lack of access, or competing priorities may prevent women from acting on that intention. Sorai Choi’s study showed that women over 50 years old were more likely to be in stage 6 and 7 (55/4%); It was consistent with the results of this study that there was a significant relationship (*p* < 0.005) between the age of performing mammography screening behavior and awareness^29^. The results of the study conducted by Seyedkanani et al. also indicated that the rate of mammography screening is higher in older women(women between the ages of 50 and 59 were 2.38 times more likely to undergo mammography than those over 60 years) which is consistent with the results of this study^1^.

Also, studies (Lee et al., 2010; Samah and Ahmadian, 2012) showed that older women are more likely than younger women to exhibit behaviors related to breast health. This may be explained by the fact that older women have access to more preventive healthcare throughout their lives, have higher levels of health literacy, and are more familiar with the importance of early detection. However, the study also suggests that younger women, especially those in lower-income or rural areas, may face barriers such as limited access to information, financial constraints, and cultural taboos, which prevent them from participating in screening. Also, the results of the study showed that there was a statistically significant relationship between the family history of breast cancer and the decision-making stages of mammography screening behavior (*p* < 0001). The study conducted by Heydari et al.^31^ also reported similar results to this study.

In our findings, awareness of breast cancer and prevention was significantly higher among participants in the decision stages of action (Stages 5 and 6), compared to those in the pre-decision stages (Stage 2 and 3). Thus, more participants who had a higher level of knowledge and awareness about breast cancer and prevention were in the stages of decision to act and action. This emphasizes the need to focus interventions on women in the early stages of decision-making to bridge the gap between awareness and action. A study conducted for cervical screening among female university students based on the precautionary acceptance process model and the health belief model in Korea by Hye Jung-shing is consistent with the results of this study^25^. Younger people who had low education and participated in the present study were less likely to have mammography screening than people with higher education, while performing mammography at a younger age and detecting breast cancer in the early stages can lead to almost complete treatment and With timely diagnosis and effective treatment, the survival rate can reach 90%.^8^

To improve screening rates, it is important to also address the cultural, financial, and geographical barriers women face. These barriers can be mitigated by increasing the availability of mobile screening units, reducing the cost of screening, and providing culturally sensitive educational materials that acknowledge specific concerns and fears regarding mammography.

## Strengths and limitations

In this study, the change of mammography screening behavior was investigated based on the decision-making stages of the precautionary acceptance process model, which has not been studied in this field so far in IRAN, and this can be considered as a strong point. However, our study has some limitations. The use of self-reported data collected through telephone interviews increases the risk of bias, as participants might provide socially desirable responses, which could distort the results. Moreover, due to the cross-sectional nature of the study, the relationships among the research variables cannot be considered as cause-and-effect relationships. Also, one of the limitations of this study is the psychological condition of people when completing the questionnaire can affect the result, which is inevitable in such cases. Our reliance on a selected cohort of consenting women limits the generalizability of the findings to the broader Iranian population. A broader retrospective analysis across the healthcare system could mitigate this limitation.

## Conclusion

The results show that variables such as age, marital status, and education level significantly influence participants’ placement in the stages of mammography screening within the PAPM model. Regression analysis revealed that individuals with lower education levels need more education to improve their awareness about mammography screening. Marital status and other demographic factors were important variables influencing participants’ decisions.

Based on the findings of this study, the Precaution Adoption Process Model is a valuable framework for planning education and health promotion in various health issues, especially mammography screening. Compared to other models like the Health Belief Model (HBM) and Transtheoretical Model (TTM), PAPM’s focus on decision-making stages offers a more personalized approach to understanding and addressing the factors influencing behavior change.

## Electronic supplementary material

Below is the link to the electronic supplementary material.

Supplementary Material 1

## Data Availability

The datasets generated and/or analyzed during the current study are not publicly available but are available from the corresponding author on reasonable request.
