# Supplementary material for: Mammography screening status of women aged 40 and older in eastern Iran using the precaution adoption process model (PAPM)
Source: Sci Rep. 2025 Jul 13;15:25329. doi: 10.1038/s41598-025-08511-3 (PMC12256610; doi:10.1038/s41598-025-08511-3)
Supplement: Supplementary file 1 — Supplementary Material 1 [file 41598_2025_8511_MOESM1_ESM.pdf]

Birjand University of Medical Sciences and Health Services

School of Health

Dear Madam,

The questionnaire provided to you is in connection with a study on breast cancer screening. Your honest cooperation in answering the questions will help advance cancer control programs. It should be noted that the questionnaire is anonymous and the information will remain confidential.

Questionnaire code…

Demographic Information

1. How old are you?

2. At what age did you get married?

3. Height...........

4. Weight..............

5. Education level: A: Elementary and Literacy Movement ☐ B: Secondary ☐ C: Diploma ☐ D: University Education ☐

6. Spouse's education level: A: Elementary and literacy movement ☐ B: Secondary school ☐ C: Diploma ☐ D: University education ☐

7. Marital status: A: Married ☐ B: Separated ☐ C: Deceased spouse ☐ D: Single ☐

8. Menopause status: A: Yes ☐ B: No ☐

9. Do you have a history of any type of breast-related disease? A: Yes ☐ B: No ☐

If yes, please state the type of disease.................................

10. Is there a history of breast cancer in your family? A: Yes ☐ B: No ☐

If yes, what is their relationship to you? ....

11. If you have been pregnant, how many pregnancies have you had so far? A: Not at all ☐ B: 1 time ☐ C: 2 times ☐ D: 3 times ☐ E: More than 3 times ☐

12. Do you have a history of smoking or using hookah? A: Yes ☐ B: No ☐

13. Have you ever used birth control pills? A: Yes ☐ B: No ☐

14. Have you ever breastfed a baby? A: Yes ☐ B: No ☐

15. Do you exercise regularly during the week? A: Yes ☐ B: No ☐

Awareness questionnaire

1. What is the purpose of a mammogram? a) Diagnosis of liver diseases ☐ b) Diagnosis of tuberculosis ☐ c) Diagnosis of breast cancer ☐ d) Don't know ☐

2. How should a mammogram be performed? A) Every 2 years after age 40 ☐ B) Every 3 years after age 40 ☐ C) Every 2 years if you have a family history of breast cancer ☐ D) Annually for the first three years and then every 3 years if the results are favorable ☐ E) I don't know ☐

3. Who is required to have a mammogram? a) Single women ☐ b) Married women ☐ c) Widowed or divorced women ☐ d) All women ☐ e) I don't know ☐

4. In which of the following cases is the risk of developing breast cancer higher?

4/1. Being overweight A: Yes ☐ B: No ☐ Don't know ☐

4/2. Pregnancy A: Yes ☐ B: No ☐ Don't know ☐

4/3. Having a family history of breast cancer A: Yes ☐ B: No ☐ Don't know ☐

4/4. Not breastfeeding the baby, A: Yes ☐ B: No ☐ Don't know ☐

4/5. Inadequate nutrition A: Yes ☐ B: No ☐ Don't know ☐

4/6. Smoking A: Yes ☐ B: No ☐ Don't know ☐

4/7. Taking birth control pills, A: Yes ☐ B: No ☐ Don't know ☐

4/8. Lack of physical activity A: Yes ☐ B: No ☐ Don't know ☐

5. Which of the following options is a sign of breast cancer? A) Any abnormal discharge from the nipple ☐ B) Inverted nipple ☐ C) Change in the appearance of the breast skin (such as orange peel) ☐ D) All of the following ☐ E) Don't know ☐

6. When is the best time to have a mammogram? A) The week before menstruation ☐ B) During menstruation ☐ C) The week after menstruation ☐ D) It can be done at any time ☐ E) I don't know ☐

7. At what age is the risk of developing breast cancer highest? A) Before 40 years of age ☐ B) Between 30 and 40 years of age ☐ C) Over 40 years of age ☐ D) Same at all ages ☐ E) Don't know ☐

8. In the early stages of breast cancer, which option is correct? A) Only detectable ☐ B) Only treatable ☐ C) Both detectable and treatable ☐ D) Neither detectable nor treatable ☐ E) I don't know ☐

9. Mammography is also performed on breasts with implants. True ☐ False ☐ Don't know ☐

10. On the day of the mammography, antiperspirant, perfume, powder, ointment, or cream should not be used. True ☐ False ☐ Don't know ☐

11. To perform a mammography, one should avoid consuming certain foods such as chocolate, coffee, and spicy spices 48 hours before it. True ☐ False ☐ Don't know ☐

12. Mammography should not be performed during menstruation. True ☐ False ☐ Don't know ☐

13. It is better to perform a mammography three months after the end of breastfeeding. True ☐ False ☐ Don't know ☐

14. There is no specific contraindication to performing a mammography during pregnancy. True ☐ False ☐ Don't know ☐

15. When is the age to start performing a mammography? A) Before 40 years of age ☐ B) After 40 years of age ☐ C) After menopause ☐ D) After marriage ☐ E) Don't know ☐

Stages determination questionnaire

1. Have you ever heard of breast cancer screening (mammography)? If your answer is yes, answer question 2. A: Yes ☐ B: No ☐

2. Have you ever had a mammogram? If your answer is no, answer question 3. A: Yes ☐ B: No ☐

3. Which of the following best describes your situation regarding having a mammogram?

3/1. I have never thought about having a mammogram. A: Yes ☐ B: No ☐

3/2. I have not yet decided whether or not to have a mammogram. A: Yes ☐ B: No ☐

3/3. I have decided not to have a mammogram. A: Yes ☐ B: No ☐

3/4. I have decided to have a mammogram. A: Yes ☐ B: No ☐
